# Spontaneous Intracranial Hypotension Manifesting Orthostatic Headache Worsen by Playing the Trombone

**DOI:** 10.7759/cureus.24577

**Published:** 2022-04-28

**Authors:** Masahito Katsuki, Shin Kawamura, Akihito Koh

**Affiliations:** 1 Department of Neurosurgery, Itoigawa General Hospital, Itoigawa, JPN; 2 Composer and Singer Song Writer, Cuty KATSKI Music Office, Niigata, JPN

**Keywords:** cerebrospinal fluied leakage, wind instrument, trauma, orthostatic headache, intracranial hypotension

## Abstract

We present the case of a 13-year-old Japanese girl with no previous medical history who presented with a gradually worsening series of orthostatic headaches. We diagnosed spontaneous intracranial hypotension, which worsened by playing the trombone and its Valsalva maneuver effect. She was admitted to our hospital and treated with rest and hydration, and discharged on day 14 without any headaches during the admission. However, her headache recurred when she played trombone, so she stopped it. She now plays percussions, and no headaches recurred for a year. Our case’s headache may have been further exacerbated by cerebrospinal fluid (CSF) leakage due to CSF pressure increase by the Valsalva maneuver while playing the trombone.

## Introduction

Intracranial hypotension typically manifests as an orthostatic headache with neck pain, dizziness, and fatigue. Orthostatic headache is affected by posture. The headache continues daily, and for which the medication is less effective. The most frequent underlying factor is cerebrospinal fluid (CSF) leakage. It is hypothesized that dural structural weakness in the dura matter may be responsible for its idiopathic or traumatic tear and consequent leakage [1]. According to The Japanese Society of Cerebrospinal Fluid Leak Official Guideline [1], traumatic intracranial hypotension is defined to have an onset of 30 days from the trauma. While spontaneous intracranial hypotension occurs more commonly in women in their late 30s, and its annual incidence is underestimated as five per 100000. The clinical manifestations are usually headache (98.5%), dizziness or vertigo (50.5%), nausea (49.0%), disequilibrium (42.6%), and posterior neck pain (34.2%). The leakage site can sometimes be identified through magnetic resonance imaging (MRI). The most common treatment is rest, hydration, or epidural patching with blood or fibrin sealant, though surgery is sometimes needed. The outcomes are usually good for most patients. However, variability in presenting signs and symptoms and low awareness of the disorder contribute to delayed diagnosis [2].

We herein describe a 12-year-old girl who presented with an orthostatic headache due to spontaneous intracranial hypotension, which is aggravated by playing the wind instrument trombone. This is one of the rare cases of headache worsened by the Valsalva maneuver due to playing the wind musical instruments [2,3].

## Case presentation

A 13-year-old Japanese girl with no previous medical history presented a gradually worsening series of orthostatic headaches for one month. There was no history of major trauma. Her orthostatic headache has a numerical rating scale of 6/10 without pulsating characteristics. The headache was not present during sitting but occurred some minutes after standing. Especially, she complained that the headache worsened when she played the trombone with both standing and sitting. There was no exacerbation of headaches due to coughing or toileting.

**Figure 1:**
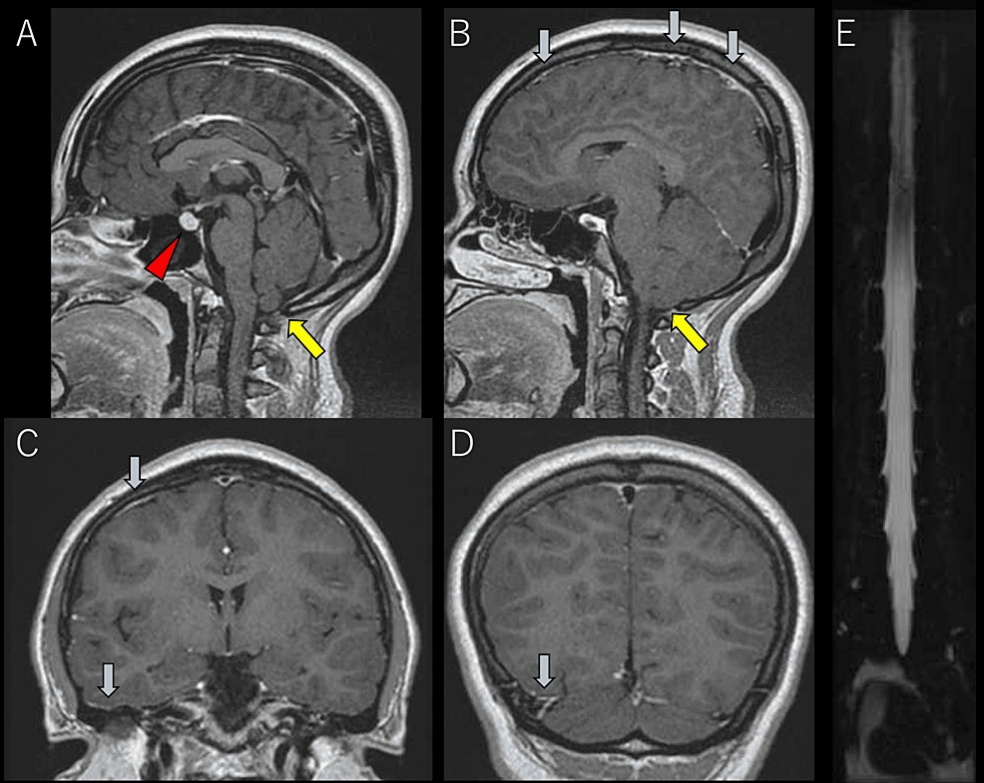
Magnetic resonance imaging The gadolinium contrast-enhanced T1-weighted magnetic resonance (MR) imaging showed partially enhanced dura matter (blue arrows in A, B, C, and D), and low-lying tonsils (yellow arrows in A and B), and pituitary enlargement (red arrowhead in A). The MR myelography could not detect findings specific to cerebrospinal fluid leakage; floating dural sac sign, incomplete floating dural sac sign, nor dinosaur tail sign (E).

The MRI showed partially contrast-enhanced dura matter (blue arrows in Figures 1A, 1B, 1C, 1D), low-lying tonsils (yellow arrows in Figures 1A, 1B), and pituitary enlargement (red arrowhead in Figure 1A). The MR myelography could not detect findings specific to CSF leakage; floating dural sac sign, incomplete floating dural sac sign, or dinosaur tail sign (Figure 1E) [1].

We diagnosed spontaneous intracranial hypotension from the medical history and the MRI findings, which was worsened by playing the trombone. We did not perform a more accurate modality for leakage localization, such as computed tomography-myelography, considering the radiation exposure and her young age. We prescribed 7.5 g of Japanese herbal Kampo medicine Goreisan to treat suidoku status (fluid disturbance such as edema, dehydration, and dislocation) via inhibiting aquaporin-4 channels [4]. The headache severity slightly improved but continued. Therefore, she was admitted to our hospital and treated with rest and hydration of 1500 mL of lactate Ringer solution, according to the treatment guidelines [1]. After a week of rest, we gradually increased the angle of lying down. The patient was discharged on day 14 without any headaches during the admission. However, her headache recurred when she played trombone, so she stopped it. She now plays percussions, and no headaches recurred for a year.

## Discussion

This is one of the rare cases of headache caused by intracranial hypotension, which was worsened by the Valsalva maneuver due to playing trombone. The expiratory pressure required to play the trombone is up to 65 cmH2O [5]. The value is not extremely vigorous, but playing musical instrument needs high expiratory pressure; clarinet for 20-55 cmH2O, saxophone 15-80 cmH2O, oboe 35-124 cmH2O, and trumpet up to 250 cmH2O [2]. Valsalva maneuver caused by these expiratory efforts seems to have a potential role in headache exacerbation. Previously, spontaneous low-pressure headaches, exacerbated by playing the bagpipe, were reported [2]. The two other cases were also reported with Chiari malformation I and hydrocephalus due to tumors whose headaches were worsened by playing cornet and horn, respectively [3]. The common mechanism in which playing wind instruments worsens the headache is that the Valsalva maneuver interferes with venous drainage. The disturbed venous drainage increases the intracranial pressure, leading to more CSF leakage or headaches. Table 1 shows the previous reports on intracranial hypotension and playing wind musical instruments.

**Table 1:** Previous reports on intracranial hypotension and playing wind musical instruments

Author	Year	Age	Sex	Instrument	Comorbidity	Symptom	Treatment
Patrick [2]	2007	40	Woman	Bagpipe	Chiari malformation I	Postural headache and neck stiffness	Lumbar 20 mL blood patch
Hernández-Palazón [3]	2013	10	Man	Cornet	Chiari malformation I	Growth retardation, headache aggravated with cough and playing cornet	Decreased his musical activity
Hernández-Palazón [3]	2013	10	Man	Horn	Hydrocephalus due to a block at the fourth ventricle outlets	Headache and vomiting for two days aggravated with playing horn	Endoscopic third ventriculostomy
Ours	2022	13	Woman	Trombone	-	Orthostatic headache	Bedrest and hydration for 14 days

Our case’s headache may have been further exacerbated by CSF leakage due to CSF pressure increase by the Valsalva maneuver while playing the trombone. We suggest that musicians who play wind musical instruments in which high expiratory pressure is required may be at the risk of intracranial hypotension exacerbation and that when such players consult their doctors regarding postural headache, the possibility of spontaneous intracranial hypotension should be considered.

The diagnostic criteria for spontaneous intracranial hypotension according to the International Classification of Headache Disorders, third edition (ICHD-3) includes; A) any headache attributed to low CSF pressure or CSF leakage that meets criterion C, B) either or both of the following: low CSF pressure as <60 mm CSF, evidence of CSF leakage on imaging, C) Headache that developed in temporal relation to the low CSF pressure or CSF leakage or that led to its discovery and D) Headache not better accounted for by another ICHD-3 diagnosis [6]. However, these findings do not necessarily appear in all the patients with intracranial hypotension. Therefore, the Japanese guidelines [1] state that if A and B are satisfied, it is considered probable intracranial hypotension; A) orthostatic headache, B) Enhancement of pachymeninges or low CSF pressure as <60 mm CSF. Our case met the Japanese guideline(s) criteria. A further consideration for diagnosing intracranial hypotension should be needed.

Data from randomized trials are not available to guide the management of spontaneous intracranial hypotension. Depending on the severity, the first treatment is a short course of conservative treatments over a few days or weeks, including bed rest, high oral fluid intake, caffeine, and an abdominal binder. If these therapies could not improve the symptoms, 1 or 2 epidural blood patches in the lumbar spine are required. A higher volume of blood patches has benefits compared to a lower volume. For patients with persistent symptoms, dynamically computed tomography-myelography or digital-subtraction myelography was used to localize the leak site and directed epidural blood patch, glue injection, or microsurgical repair are considered [6]. Also, in the Japanese guidelines [1], the first choice of treatment is two weeks of bed rest and hydration. After that, an epidural blood patch is considered. We performed 14 days of admission with bed rest and hydration, and the symptoms improved.

## Conclusions

We experienced the case with an orthostatic headache due to intracranial hypotension exacerbated by the Valsalva maneuver while playing trombone. She did not have any history of major trauma, so we diagnosed spontaneous intracranial hypotension. When we see musicians who play wind musical instruments with orthostatic headaches affected by their postures, the possibility of spontaneous intracranial hypotension should be considered.
